# Sitagliptin Modulates the Response of Ovarian Cancer Cells to Chemotherapeutic Agents

**DOI:** 10.3390/ijms21238976

**Published:** 2020-11-26

**Authors:** Agnieszka Kosowska, Wojciech Garczorz, Agnieszka Kłych-Ratuszny, Mohammad Reza F. Aghdam, Małgorzata Kimsa-Furdzik, Klaudia Simka-Lampa, Tomasz Francuz

**Affiliations:** Department of Biochemistry, Faculty of Medical Sciences in Katowice, Medical University of Silesia, Medyków 18, 40-752 Katowice, Poland; wojtekg@sum.edu.pl (W.G.); aklych@sum.edu.pl (A.K.-R.); reza_aghdam@hotmail.com (M.R.F.A.); malgorzata.kimsa@sum.edu.pl (M.K.-F.); ksimka@sum.edu.pl (K.S.-L.); tfrancuz@sum.edu.pl (T.F.)

**Keywords:** sitagliptin, ovarian cancer, diabetes, metastasis, metalloproteinases, apoptosis

## Abstract

The strong association between diabetes mellitus type 2 and cancer is observed. The incidence of both diseases is increasing globally due to the interaction between them. Recent studies suggest that there is also an association between cancer incidence and anti-diabetic medications. An inhibitor of dipeptidyl-peptidase 4 (DPP-4), sitagliptin, is used in diabetes treatment. We examined the influence of sitagliptin alone or in combination with a cytostatic drug (paclitaxel) on the development of epithelial ovarian cancer cells and the process of metastasis. We examined migration, invasiveness, apoptosis, and metalloproteinases (MMPs) and their inhibitors’ (TIMPs) production in two human ovarian cancer cell lines. Sitagliptin induced apoptosis by caspase 3/7 activation in paclitaxel-treated SKOV-3 and OVCAR-3 cells. Sitagliptin maintained paclitaxel influence on ERK and Akt signaling pathways. Sitagliptin additionally reduced migration and invasiveness of SKOV-3 cells. There were distinct differences of metalloproteinases production in sitagliptin-stimulated ovarian cancer cells in both cell lines, despite their identical histological classification. Only the SKOV-3 cell line expressed MMPs and TIMPs. SKOV-3 cells co-treated with sitagliptin and paclitaxel decreased concentrations of MMP-1, MMP-2, MMP-7, MMP-10, TIMP-1, TIMP-2. The obtained data showed that sitagliptin used with paclitaxel may be considered as a possibility of pharmacological modulation of intracellular transmission pathways to improve the response to chemotherapy.

## 1. Introduction

The global statistics demonstrate the epidemic nature of diabetes. Interestingly, both diabetes and cancer are prevalent diseases whose incidence is increasing globally due to the interaction between them [1]. Ovarian cancer is one of the most common cancers affecting women and is characterized by a very high mortality rate. Furthermore, studies have shown that patients manifesting both diseases have worse prognosis, higher mortality, and shorter relapse-free survival [2].

In the treatment of diabetes, gliptins drugs are commonly used. Sitagliptin is the first selective inhibitor of dipeptidyl-peptidase 4 (DPP-4) which belongs to gliptins groups. This drug was indicated for the treatment of type 2 diabetes mellitus (DMT2) to improve glycemic control in combination with metformin [3]. Dipeptidyl-peptidase 4 inhibitors can reduce the enzyme activity up to 70–90%. DPP-4 is an enzyme that inactivates the two incretin hormones: Glucagon-like peptide-1 (GLP-1) and glucose-dependent insulinotropic polypeptide. Incretin hormones stimulate insulin secretion from the β-cells and inhibit glucagon secretion from the α-cells [4]. Sitagliptin inhibits DPP-4 activity; therefore, it prolongs the incretin hormones’ action. As a result, substantial stimulation of insulin secretion and suppression of glucagon secretion postprandially are observed [5,6]. Sitagliptin does not have a direct effect on satiety or gastric emptying. Moreover, it decreases the level of free fatty acids and thereby also has insulin-sensitizing properties [7]. Furthermore, sitagliptin was shown to have potent anti-inflammatory properties by suppressing the expression of pro-inflammatory genes [8].

It is estimated that the majority of ovarian cancers develop in the epithelium. Epithelial ovarian cancer is divided into several subtypes, for instance: Serous, endometrioid, clear cell, mucinous, and mixed [9]. Ovarian cancer can also originate from germ cells or stromal tissue; however, these types of ovarian cancer are diagnosed at earlier stages on average, and therefore have much higher survival rates. Another type, small cell carcinoma of the ovary, is a hypercalcemic untreatable type of rare ovarian cancer, typically found in young women [10].

The reason for the high mortality rate of the most common epithelial ovarian cancer (EOC) is the lack of specific clinical symptoms at an early stage. Seventy percent of patients are diagnosed at advanced stages (stages III–IV) with disseminated intra-abdominal metastasis [11].

The standard treatment for EOC uses carboplatin and paclitaxel [12]. Weekly treatment with paclitaxel may exploit anticancer mechanisms such as induction of apoptosis or angiogenesis inhibition [13]. Clinical trials data showed that this treatment is used in the majority of ovarian cancers. However, more than 70% of patients suffer from recurrence which increases chemoresistance [14]. Due to the absence of effective treatment for advanced stage or recurrence, the high mortality rate of ovarian cancer requires new therapeutic modalities.

*In vitro* studies on endometrial cancer cell lines have shown that metformin, which is an anti-diabetic drug, enhanced apoptosis of endometrial cancer cells in the presence of paclitaxel [15]; however, at this moment there have been no data presenting the influence of sitagliptin on the development of EOC and the process of metastasis.

Cancer metastasis is enabled by the presence of proteolytic enzymes called matrix metalloproteinases (MMPs) [16,17]. MMPs’ activity is under the control of tissue inhibitors of metalloproteinases (TIMPs). In the state of imbalance between the concentration of MMPs and their inhibitors, an intensified metastasis process is observed. There are 23 members in the MMP family; many of them have been reported to be associated with ovarian cancer. Ovarian tumor cells and the surrounding stromal cells stimulate the synthesis or activation of various MMPs to aid tumor growth, invasion, and eventual metastasis [18]. It has been reported that overexpression of MMPs is associated with the increased metastasis in ovarian tumors, which results in poor prognosis and decreases survival. However, the pattern expression of different MMPs depends on the type of a tumor or tumor stage.

The effective angiogenesis is necessary for tumor growth and the process of metastasis. A tumor without adequate vascularization undergoes either necrosis or apoptosis [19]. The promoters of angiogenesis are induced by interactions of VEGF, FGF1 and 2, PDGF, EGF, and their specific receptors [20]. Epithelial ovarian cancer cells commonly metastasize through the transcoelomic route. Tumor infiltrates directly into the neighboring organs. EOC primarily disseminates to the omentum and peritoneum. During intravasation, tumor cells interact with endothelium directly [21]. Endothelial cells, which are the inner layer of blood vessels of the tumor stromal tissue, are the main targets of anti-angiogenic therapy [22]. Our previous study showed that incretins used in the treatment of diabetes inhibit activation of apoptosis in TNF-α-stimulated endothelial cells, and reduce the expression of MMPs (MMP-1 and MMP-9) which are relevant for tumor development [23].

However, the molecular mechanisms underlying ovarian cancer metastasis still remain unelucidated. Due to the suggested association between cancer and diabetes, we wonder if an anti-diabetic drug, sitagliptin, can be used in the treatment of type 2 diabetes in patients with co-existing ovarian cancer.

## 2. Results

### 2.1. Distinct Metalloproteinases Production in Sitagliptin Stimulated Ovarian Cancer Cells

**Figure 1:**
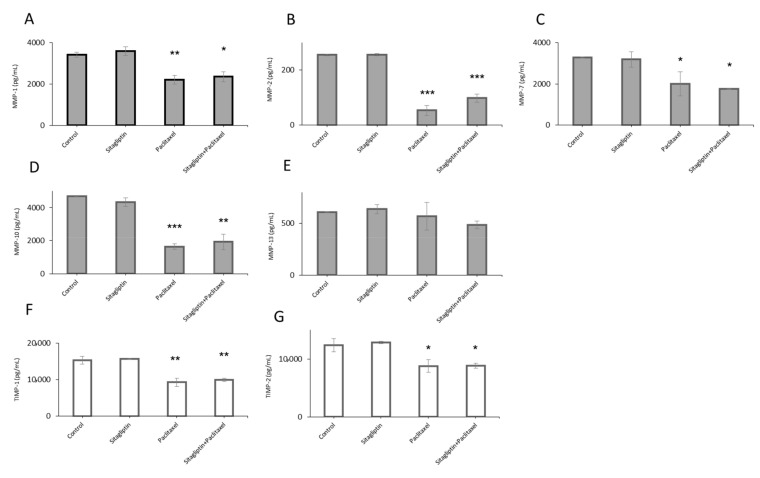
Metalloproteinase (MMP) and metalloproteinase inhibitors’ (TIMP) profiles in SKOV-3 after sitagliptin (50 µM), paclitaxel (10 nM) or sitagliptin (50 µM) and paclitaxel (10 nM) treatment. Protein levels of MMP-1 (**A**), MMP-2 (**B**), MMP-7 (**C**), MMP-10 (**D**), MMP-13 (**E**), TIMP-1 (**F**), and TIMP-2 (**G**) in cell medium were analyzed using multiplex analysis after 24-h incubation with tested substances. Mean values ± SD are shown. *n* = 2–3 per group. * *p* < 0.05, ** *p* < 0.01, *** *p* < 0.001. Control vs. different conditions. One-way ANOVA followed with Dunnett’s *post hoc*.

Ovarian cancer cells, SKOV-3 and OVCAR-3, were incubated with sitagliptin (50 µM) and paclitaxel (10 nM) or both, then MMP and TIMP levels were evaluated. MMP-1, MMP-2, MMP-7, MMP-9, MMP-10, MMP-13, TIMP-1, and TIMP-2 protein concentrations in culture cell medium were assessed upon stimulation in both cell lines. Only detectable levels of MMPs and TIMPs were observed in the SKOV-3 cell line, whereas MMP and TIMP levels were under detection limits in OVCAR-3. After treatment with sitagliptin, there were no significant changes in MMP and TIMP concentrations. Upon paclitaxel stimulation and sitagliptin with paclitaxel stimulation the concentrations of MMPs were decreased: MMP-1 by 36% and 31%, respectively (Figure 1A), MMP-2 by 79% and 61%, respectively (Figure 1B), MMP-7 by 39% and 46%, respectively (Figure 1C), and MMP-10 by 65% and 59%, respectively (Figure 1D). Furthermore, the concentrations of MMPs inhibitors decreased: TIMP-1 by 40% and 35%, respectively, for paclitaxel and sitagliptin with the paclitaxel group (Figure 1F), and TIMP-2 by 29% and 29%, respectively (Figure 1G). There were no significant changes in MMP-13 concentration after the stimulation (Figure 1E).

### 2.2. Sitagliptin Reduces Migration and Invasiveness of Ovarian Cancer Cells

**Figure 2:**
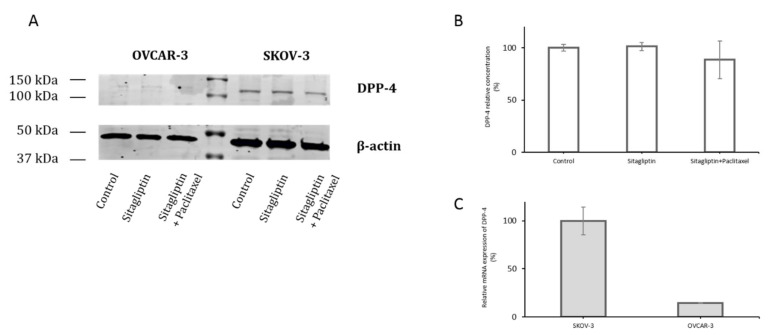
Dipeptidyl-peptidase 4 (DDP-4) (CD26) western blot and mRNA expression in ovarian cancer cells. Representative western blot image of protein extracts from OVCAR-3 and SKOV-3 (**A**). SKOV-3 cell line—control, cells stimulated for 24 h with sitagliptin (50 µM), sitagliptin (50 µM), and paclitaxel (10 nM). DPP-4 densitometry normalized protein concentration (**B**). Relative mRNA expression of *DPP-4* assessed by qPCR (**C**).

The main effect of sitagliptin is achieved by inhibiting DPP-4. In SKOV-3 and OVCAR-3 cell lines, mRNA for *DPP-4* was detected, but in OVCAR-3, this level was 7 times lower than in SKOV-3 (Figure 2C). The presence of DPP-4 was shown in western blot only in the SKOV-3 cell line (Figure 2A,B).

**Figure 3:**
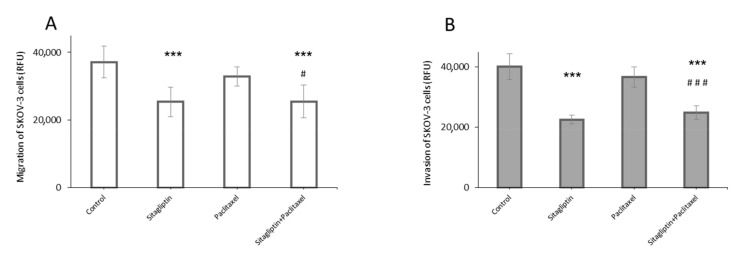
Sitagliptin effects on migration and invasion of ovarian cancer cells. SKOV-3 cell line was stimulated for 24 h with sitagliptin (50 µM) and paclitaxel (10 nM) or both for transwell migration assay (**A**) and transwell invasion assay (**B**). Relative fluorescence unit (RFU) was measured. Mean values ± SD are shown. *n* = 6 per group. *** *p* < 0.001 control vs. different conditions. ^#^ *p* < 0.05, ^###^ *p* < 0.001 paclitaxel vs. sitagliptin + paclitaxel group. One-way ANOVA followed with Tukey’s *post hoc*.

In transwell-based migration assay, sitagliptin reduced migration of SKOV-3 cell (Figure 3A). Sitagliptin (50 µM) was added into the upper chamber and the amount of cell migration was reduced by 32% in SKOV-3 cells. Additionally, treatment of paclitaxel (10 nM) did not influence on migration, but co-treatment sitagliptin with paclitaxel maintained the migration potential reduced by 31% compared to the control in SKOV-3 cells. Furthermore, the transwell invasion assay, a more sophisticated model of invasion, was developed. Ovarian cancer cells must migrate through the layer of human lung microvascular endothelial cells (HMVEC) and through the Matrigel, which simulates the natural environment of tumor invasion. Such a model was prepared, and it was observed that sitagliptin reduced the invasion of SKOV-3 cells. Sitagliptin (50 µM) was added into the upper chamber and the number of cells that migrated through the layer of endothelial cells, Matrigel, and 8-μm transwell filters, was reduced by 44%. In addition, treatment of paclitaxel (10 nM) did not influence invasion, but co-treatment sitagliptin with paclitaxel maintained the invasion potential reduced by 38% compared to the control in SKOV-3 cells (Figure 3B).

### 2.3. Sitagliptin Induces Caspase Activity in Paclitaxel Stimulated Ovarian Cancer Cells

**Figure 4:**
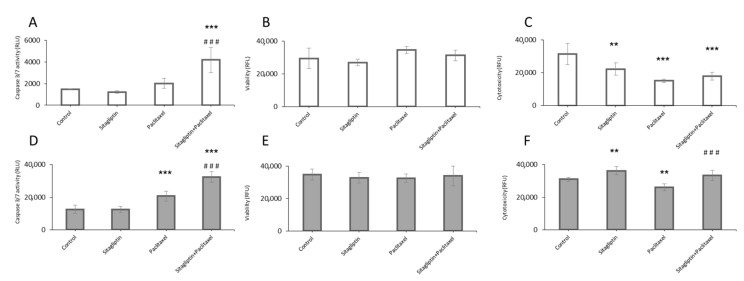
Sitagliptin effects on apoptosis, cell viability, and cytotoxicity. Ovarian cancer cell lines were stimulated for 24 h with sitagliptin (50 µM) and paclitaxel (10 nM) or both in SKOV-3 (**A**–**C**) and OVCAR-3 (**D**–**F**). Caspase 3/7 activation in SKOV-3 (**A**), cell viability in SKOV-3 (**B**), and cytotoxicity in SKOV-3 (**C**) was analyzed. Caspase 3/7 activation in OVCAR-3 (**D**), cell viability in OVCAR-3 (**E**), and cytotoxicity in OVCAR-3 (**F**) was analyzed. Relative fluorescence unit (RFU) or relative luminescence unit (RLU) was measured. Mean values ± SD are shown. *n* = 6 per group. ** *p* < 0.01, *** *p* < 0.001 control vs. different conditions. ^###^ *p* < 0.001 paclitaxel vs. sitagliptin + paclitaxel group. One-way ANOVA followed with Tukey’s *post hoc*.

To assess the influence of sitagliptin and paclitaxel on apoptosis, cell viability, and cytotoxicity, SKOV-3 and OVCAR-3 cells were stimulated with: Sitagliptin at 50 µM for 24 h with or without paclitaxel at 10 nM or paclitaxel alone (Figure 4). In both analyzed cancer cell lines, the treatment with sitagliptin caused no significant changes in caspase 3/7 activity. Paclitaxel induced the activation of caspase 3/7 by 65% in OVCAR-3. The co-treatment with sitagliptin and paclitaxel further increased activation of caspases by 160% in OVCAR-3 and 180% in SKOV-3 as compared to the control (Figure 4A,D). However, in both ovarian cancer cell lines, stimulation with sitagliptin (50 µM) and paclitaxel (10 nM) caused no significant changes in viability (Figure 4B,E). Interestingly, the cytotoxicity effect of tested drugs was different in SKOV-3 and OVCAR-3. In SKOV-3, both sitagliptin and paclitaxel caused a decrease in cytotoxicity (Figure 4C). In OVCAR-3, an increased cytotoxic effect was observed in cells treated with sitagliptin (by 16%) compared to the control (Figure 4F).

**Figure 5:**
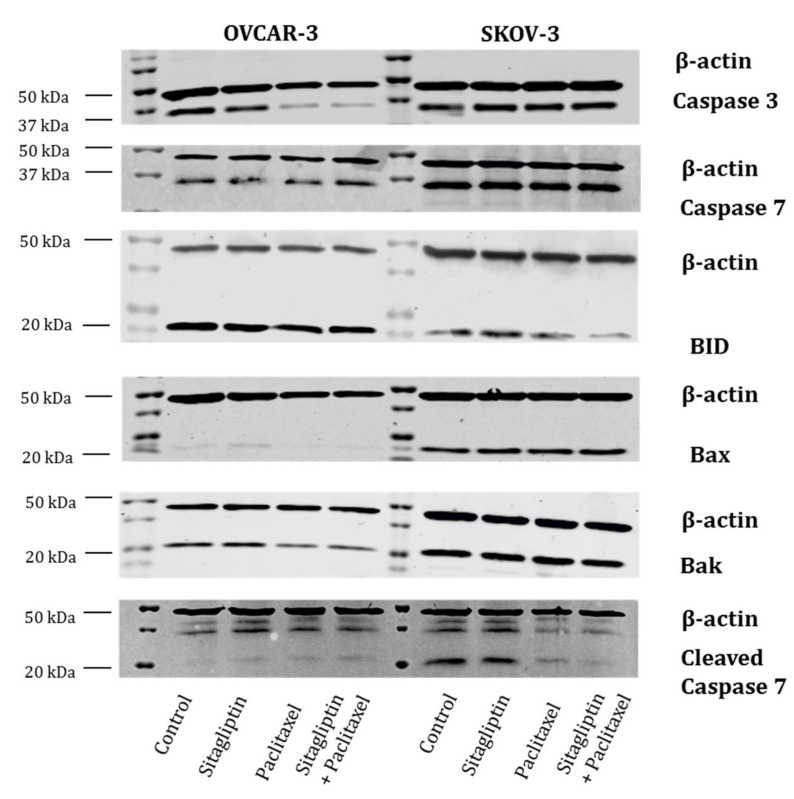
Western blots. Caspase 3, caspase 7, BID, Bax, Bak, cleaved caspase 7. Representative western blot image of protein extracts from OVCAR-3 and SKOV-3. Ovarian cancer cell lines: Control cells, cells stimulated for 24 h with sitagliptin (50 µM), paclitaxel (10 nM), or sitagliptin (50 µM) and Paclitaxel (10 nM).

In parallel, the presence of apoptotic proteins was tested by western blot. A comparison between two analyzed cell lines was completed. Once again, there were differences observed between ovarian cancer cell lines (Figure 5). The presence of caspase 3, caspase 7, and BID was detected in both SKOV-3 and OVCAR-3. While the proteins of Bax, Bak, and cleaved caspase 7 were present in SKOV-3, in OVCAR-3 there were only traces of these proteins.

### 2.4. Sitagliptin Maintained Paclitaxel Influence on ERK Signaling Pathway by Inhibition of Phosphorylation on Residue Thr185 and Thr187

**Figure 6:**
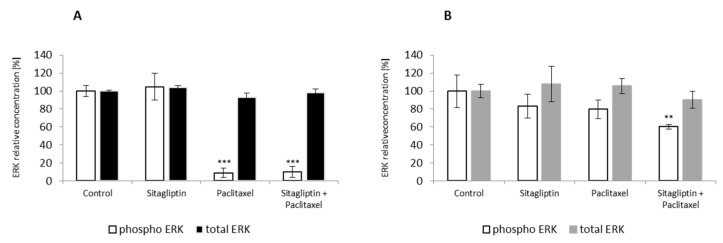
Total and phosphorylated at Thr185 and Thr187 ERK kinase after sitagliptin (50 µM), paclitaxel (10 nM), or sitagliptin (50 µM) and paclitaxel (10 nM) treatment. SKOV-3 cell line (**A**) and OVCAR-3 cell line (**B**) were stimulated for 24 h. Total and phosphorylated ERK1/2 (Thr185/Thr187) were analyzed with Luminex assay in cell lysates. Mean fluorescence intensity (MFI) was measured and recalculated relative to the control values. Mean values ± SD are shown. *n* = 3 per group. *** *p* < 0.001, ** *p* < 0.01 control vs. different conditions. One-way ANOVA followed with Dunnett’s *post hoc*.

To determine whether sitagliptin has any effect on ERK signaling pathway, SKOV-3 and OVCAR-3 were stimulated for 24 h with sitagliptin at 50 µM and paclitaxel at 10 nM or both drugs. Paclitaxel alone and sitagliptin with paclitaxel inhibited phosphorylation of ERK at Thr185 and Thr187 residues in SKOV-3 cells. Particularly, paclitaxel inhibited ERK phosphorylation by 91%, and in the sitagliptin co-treated group the inhibition was by 90% (Figure 6A) compared to the control. However, in OVCAR-3 cells, sitagliptin with paclitaxel inhibited phosphorylation of ERK at Thr185 and Thr187 residues by 39.5% compared to the control (Figure 6B).

### 2.5. Sitagliptin Maintained Paclitaxel Influence on Akt Signaling Pathway by Inhibition of Phosphorylation on Residue Thr308 and Ser473

**Figure 7:**
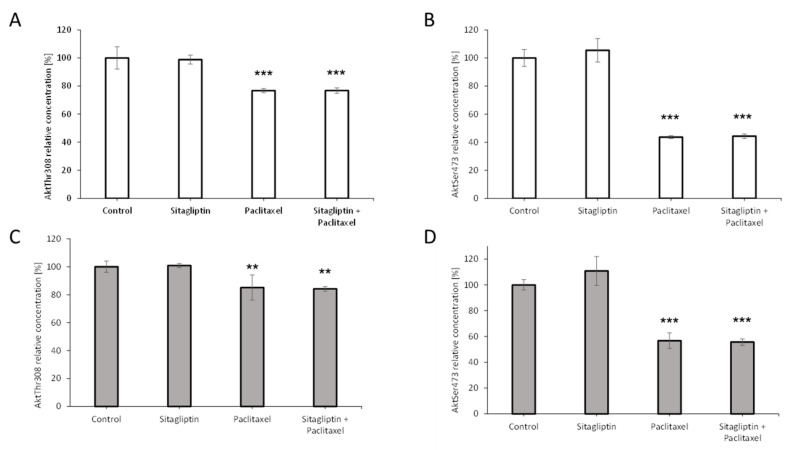
Phosphorylation of Akt kinase at Thr308 and Ser473 after sitagliptin (50 µM), paclitaxel (10 nM), or sitagliptin (50 µM) and paclitaxel (10 nM) treatment. Phosphorylation of Akt at Thr308 in SKOV-3 (**A**), phosphorylation of Akt at Ser473 in SKOV-3 (**B**), phosphorylation of Akt at Thr308 in OVCAR-3 (**C**), phosphorylation of Akt at Ser473 in OVCAR-3 (**D**), in cell lysates analyzed by PathScan Intracellular Signaling Array Kit after 24-h incubation with tested substances. Mean values ± SD are shown. *n* = 4 per group. ** *p* < 0.01, *** *p* < 0.001. Control vs. different conditions. One-way ANOVA followed with Dunnett’s *post hoc*.

To determine the effect of sitagliptin on Akt signaling pathway, SKOV-3 and OVCAR-3 were stimulated for 24 h with sitagliptin at 50 µM and paclitaxel at 10 nM or both drugs. Paclitaxel alone and sitagliptin with paclitaxel inhibited phosphorylation at the Thr308 residue and Ser473 residue in both analyzed cell lines. Particularly, in SKOV-3 cells the inhibition of Akt phosphorylation at the Thr308 residue by 23% in both groups was observed, respectively (Figure 7A). Similar inhibition of Akt phosphorylation at the Ser473 residue by 56% and 55% in paclitaxel and sitagliptin with paclitaxel groups was observed, respectively (Figure 7B). In the second ovarian cell line, OVCAR-3, the inhibition of Akt signaling pathway was also observed. Paclitaxel alone and sitagliptin with paclitaxel inhibited phosphorylation at Thr308 by 15% and 16%, respectively (Figure 7C) and at the Ser473 residue by 43% and 44%, respectively (Figure 7D).

## 3. Discussion

Epidemiological evidence indicates that diabetes type 2 is associated with the increased risk of several cancers, including ovarian cancer. Novel antidiabetic drugs are DPP-4 inhibitors [24]. The mechanism of production and degradation of incretin hormones must be taken into consideration while understanding the biology of DPP-4. Moreover, DPP-4 is overexpressed in a number of metabolic diseases (including diabetes, obesity, cardiovascular disease) or cancer [5]. Thus, the investigation of new possibilities and expanding opportunities for prevention and treatment of diabetes and coexisting diseases is crucial [25]. DPP-4 inhibitors have been shown to influence cancer cells by altering proliferation, apoptosis, extracellular matrix remodeling, and the response to chemotherapy. Due to its pleiotropic potential, this research is focusing on understanding whether sitagliptin, a DPP-4 inhibitor, has a positive or negative influence on tumor progression. The data gathered by Wenjing et al. demonstrated that exendin-4, an incretin mimetic drug, inhibits growth and enhances apoptosis of ovarian cancer cells through inhibition of the PI3K/Akt pathway [26]. Similar results were found by Kosowska et al., demonstrating that exendin-4 can reduce the cancer cell proliferation and dissemination potential, and limit the risk of metastasis, through modulation of ovarian cancer and endothelial cell interaction [23]. However, the influence of DPP-4 inhibitors was not investigated yet in ovarian cancer cells. In this study, several aspects of ovarian cancer progression were analyzed, including viability, cytotoxicity, apoptosis, migration, invasiveness, production of metalloproteinases, and their inhibitors.

In ovarian cancer treatment, the combination of paclitaxel with carboplatin showed an improvement in progression-free survival and overall survival of patients with recurrent ovarian cancer [27]. Unfortunately, the optimal management of recurrent ovarian cancer (ROC) still remains uncertain. An estimated 85% of patients with epithelial ovarian cancer who achieve a full remission following first-line therapy will develop recurrent disease with median survival from one to two years. For such patients, the aim of therapy should be focused on palliative care, maintenance of quality, and extension of life, especially those with coexisting diabetes [28]. Broekman et al. showed that the combination of metformin with carboplatin and paclitaxel was tolerable and no dose-limiting toxicities occurred in diabetic patients [29]. The usage of metformin has been correlated with enhanced progression-free survival in ovarian cancer patients. Moreover, the response of ovarian cancer cells was not homogeneous to carboplatin or paclitaxel treatment [30].

In this study, the sitagliptin alone or in combination with paclitaxel was used to assess the influence of the DPP-4 inhibitor on ovarian cancer cells. There were significant differences between the analyzed ovarian tumor cell lines. The sitagliptin affected both cell lines either with low or high invasive potential. Sitagliptin modulated the production of proteins involved in the process of apoptosis, as well as reduced the synthesis of proteins involved in the metastasis process. Interestingly, the cytotoxic effect of tested drugs was different in both analyzed cell lines, despite the identical classification of epithelial cancer. In cells with high potential of invasiveness, SKOV-3 with both sitagliptin and paclitaxel decreased a cytotoxic effect in used concentrations. Whereas in OVCAR-3, the enhanced cytotoxic effect was observed in groups treated with sitagliptin and sitagliptin with a cytostatic drug. The other diversities were observed while analyzing the apoptosis process in given cells. The trend in activation of caspases was the same in both cell lines; however, the relative fluorescence units expressing basal activity of apoptotic enzymes in SKOV-3 were ten times lower than in OVCAR-3. The interesting observation was done, while analyzing the effective caspases 3/7. Co-treatment with sitagliptin and paclitaxel triggered activation of these caspases inducing a stronger apoptosis process than paclitaxel alone, but the production of apoptotic proteins was different in SKOV-3 and OVCAR-3. It suggested that the sitagliptin induced the apoptosis process via different molecular pathways in the analyzed cancer cells, influencing in a slightly different way in each ovarian cancer cell line. Studies showed that another antidiabetic drug, metformin, acts through the Akt signaling pathway [30,31]. In our study, we analyzed the influence of sitagliptin on both ERK and Akt signaling pathways. The Akt signaling pathway is involved in MMPs expression in various cells. Both ERK and Akt pathways are involved in the migration of cancer cells [32,33]. A previous study showed that paclitaxel induces ovarian tumor cell apoptosis via the TNF-induced ERK/Akt signaling pathway. The sitagliptin maintained the effect obtained in cells treated with paclitaxel in Akt and ERK pathways in SKOV-3 and OVCAR-3 cells. The mechanism of action of sitagliptin should be further investigated, because it seems to involve other signal pathways as well. The observed heterogeneity emphasizes the key feature of this tumor, explaining, in part, the lack of successful treatment. Such heterogeneity represents a weak point of many cancers, including ovarian cancer [34]. EOCs often present complex morphology with mixed patterns and contingents of different tumor grades [35]. That is why it is so important to implement genotyping technologies which would help to detect the complexity of EOC and focus on therapy of specific tumorigenic pathways and targetable biomarkers. The tumor heterogeneity requires the usage of new technologies based on gene sequencing, for instance NGS or even single-cell RNA sequencing. This would allow for patients with distinct therapeutic vulnerabilities to be identified and use specific personalized drugs [36,37].

Extracellular matrix (ECM) stabilizes typical tissue morphology. The structure and biochemical diversity of the ECM make it a significant regulator of cell behavior. ECM proteins, as well as proteases like MMPs, are involved in ovarian tumor progression and metastasis. Metalloproteinases, for example MMP-2 and MMP-9, are highly expressed in tumors, and it is confirmed that the overexpression is associated with decreased survival rates [38]. In addition to promoting invasion by remodeling the ECM, MMP-9 also facilitates angiogenesis and cell adhesion in ovarian cancer [22]. Although the expression of MMPs and TIMPs is an individual feature, it depends not only on the tumor type, but also on the stage of tumor or race and ethnicity [39]. Both investigated cell lines were epithelial type of cancer tumors. While in OVCAR-3 we were not able to assess detectable protein levels of MMPs nor TIMPs, the SKOV-3 was characterized by very potent production of proteins: MMP-1, MMP-7, MMP-10, TIMP-1, TIMP-2, and mean production of MMP-2 and MMP-13. Furthermore, we have shown that sitagliptin alone has no influence on MMP/TIMP profiles, while when it was used with paclitaxel, it maintained the chemotherapeutic effect, observed in the reduced MMPs’ protein production. The process of cell migration is a crucial feature of living cells, as well as a critical factor for cell development or metastasis and inflammation. In this study, the methods measuring tumor cell migration and invasion were used. The transwell cell migration and invasion assays that measure the capacity of cell motility and invasiveness toward a chemoattractant gradient are more sophisticated than a simple model of the scratch assay [40]. For those assays, the SKOV-3 cells were chosen, because they possess the high migration potential and the MMPs and TIMPs proteins were detected in pre-study. It was shown that sitagliptin reduced migration and invasion in SKOV-3 cells, and this phenomenon was also observed after adding a chemotherapeutic agent, paclitaxel. However, the production of cellular metalloproteinases by SKOV-3 cells was reduced after stimulation with sitagliptin and paclitaxel. Those results suggested that the mechanism of migration and invasion of SKOV-3 were not involved only enzymes degrading extracellular matrix, but probably also required dynamic adhesion structures, invadopodia [41].

Based on the results of the study and a biological diversity of ovarian cancer cells, it could be concluded that sitagliptin in combination with paclitaxel might be a safe therapy in people with diabetes and ovarian cancer. Additionally, the observed intensification of the apoptosis process in both analyzed cancer cell lines expressed as caspase 3/7 activity was shown to support this thesis. The differences in the production of executive proteins involved in the process of apoptosis suggest that sitagliptin increases the process of cell apoptosis; however, it also activates other intracellular signaling pathways.

The obtained data might be an interesting premise for conducting further studies on the possibility of pharmacological modulation of intracellular transmission pathways to improve the response to chemotherapy. Hence, this study will create a better understanding of the influence of antidiabetic drugs on human cancer progression, which might have enormous practical significance and might be the assumption for the modification of diabetes therapy in women with ovarian cancer. The impact of gliptins on ovarian cancer cells would be a strong support for the thesis that this antidiabetic therapy in patients with ovarian cancer is safe. Of course, such a conclusion requires prospective investigations on animal models before clinical trials. A better understanding of the biology of the tumor and the development of new therapeutics requires precise murine model, established for EOC. Animal models still remain a major source of *in vivo* information. However, only a few of the potential anticancer compounds that have been evaluated in animal models have demonstrated sufficient clinical activity in phase III clinical trials [42]. These limitations highlight the necessity of improved preclinical models with an adequate pattern of tumor growth and metastases that occur in the patient and recapitulate the patient’s response to therapeutics.

## 4. Materials and Methods

### 4.1. Cell Lines

Human ovarian cancer cell lines, SKOV-3 and OVCAR-3, were purchased from American Type Tissue Culture Collection (ATCC, Manassas, VA, USA). Cells were cultured at 37 °C in 5% CO_2_ humidified atmosphere in either McCoy’s 5A modified medium with 10% FBS and 1% antibiotics or RPMI-1640 medium with 20% FBS, 1% antibiotics, and 0.01 mg/mL bovine insulin, respectively. Unless otherwise stated, all reagents were purchased from Merck KGaA, Darmstadt, Germany.

Human lung microvascular endothelial cells (HMVEC) were purchased from Lonza and were cultured in EBM-2 culture medium supplemented with EGM-2 MV SingleQuots (Lonza, Basel, Switzerland).

### 4.2. Immunoblotting

Ovarian cancer cells were seeded into 6-well plates and maintained in the appropriate medium until confluence. Subsequently, cells were incubated for 24 h with sitagliptin (50 µM) and paclitaxel (10 nM). Immediately after incubation, cells were rinsed twice with PBS buffer and lysed with PathScan cell lysis buffer (Cell Signaling, Danvers, MA, USA) supplemented with phenylmethylsulfonyl fluoride (PMSF) and protease inhibitors (Roche, Burgess Hill, UK). Total protein concentration was determined with bicinchoninic acid method. Equal amounts of protein were loaded and separated by SDS-PAGE, followed by transfer onto low autofluorescence immobilon-FL PVDF membrane (Millipore, Burlington, MA, USA). Membranes were incubated overnight at 4 °C with primary rabbit antibodies against DPP-4, caspase 3, caspase 7, BID, Bax, Bim, Bak, and cleaved caspase 7 (Cell Signaling, Danvers, MA, USA), all at 1:1000 dilution and mouse β-actin 1:5000. The signal was detected with fluorescent dye IRDye800 (LI-COR Biosciences, Lincoln, NE, USA) conjugated goat anti-rabbit secondary antibody and IRDye700 goat anti-mouse secondary antibody, and visualized by using the LICOR Odyssey Infrared Imaging System. Densitometric data were normalized to β-actin. Image acquisition and analysis were performed on the Image Studio Lite 5.2.5 platform (LICOR).

### 4.3. Fluorescent Bead-Based Luminex Cytokine Assay and MAP Kit

MMP and TIMP protein concentrations were measured using multiplex, bead-based (Luminex) assays on a Bio-Plex 200 suspension array system in accordance with each manufacturer’s instructions. The Bio-Plex Pro Human MMP 9-Plex Panel and Bio-Plex Pro Human TIMP 4-Plex Panel (Bio-Rad Laboratories, Watford, UK) were used for detection of MMPs and TIMPs in cell medium. Total and Phospho ERK protein concentrations were measured in cell lysates using Milliplex MAP Kit (2-Plex Total/Phospho ERK, Merck KGaA, Darmstadt, Germany). Data were acquired on a validated and calibrated Bio-Plex 200 system (Bio-Rad Laboratories, Watford, UK) and analyzed with Bio-Plex Manager 6.0 software (Bio-Rad Laboratories, Watford, UK) with a detection target of 50 beads per region, low RP1 target for CAL2 calibration, and recommended doublet discriminator (DD) gates of 5000–25,000 for Bio-Plex. The median fluorescence intensity (MFI) was measured.

### 4.4. Intracellular Signaling Assay

Ovarian cancer cells were cultured on 6-well cell culture plates and maintained until confluence. In the next step, cells were co-cultured with tested substances: Sitagliptin (50 µM) and paclitaxel (10 nM) or both drugs for 24 h. After incubation cells were lysed with PathScan Sandwich ELISA Lysis Buffer (Cell Signaling, Danvers, MA, USA) supplemented with phenylmethylsulfonyl fluoride (PMSF) and protease inhibitors (Roche, Burgess Hill, UK). Obtained cell lysates were used to determine the protein concentration with the bicinchoninic acid method and then the PathScan Intracellular Signaling Array Kit (Cell Signaling, Danvers, MA, USA) was used for the simultaneous detection of signaling molecules in accordance with the manufacturer’s instructions.

### 4.5. Viability, Cytotoxicity, and Apoptosis Assays

The ApoTox-Glo Triplex assay (Promega, Madison, WI, USA) was performed to assess viability, cytotoxicity, and caspase-3/7 activation within a single assay well. Ovarian cancer cells were seeded into a 96-well plate at a total density of 1 × 10^4^ cells per well in appropriate cell culture medium. Subsequently, cells were incubated for 24 h with sitagliptin (50 µM) and paclitaxel (10 nM). The analysis was performed in accordance with the manufacturer’s protocol. Briefly, after 24 h, the viability/cytotoxicity reagent, containing both the GF-AFC substrate and the bis-AAF-R110 substrate, was added to each well and incubated for 30 min at 37 °C. Fluorescence was measured at a 400ex/505em (viability) and 485ex/520em (cytotoxicity) using a microplate reader Infinite M200 (TECAN, Männedorf, Switzerland). Next, the caspase-Glo 3/7 reagent was added to the wells and incubated for 30 min at room temperature. Luminescence proportional to the caspase activity was measured using Infinite M200.

### 4.6. Transwell Migration Assay

Ovarian cancer cells migration was performed using an 8-μm transwell filters (Greiner Bio-One, Kremsmünster, Austria) in a 24-well plate format, as previously described [23]. For the migration assay, 2.5 × 10^5^ cells were placed in the upper compartment of the transwell in 200 μL medium (0.5% FBS), sitagliptin (50 µM), paclitaxel (10 nM). Cells were induced to actively migrate through membrane into the lower compartment containing 600 μL of medium and 0.5% FBS. Cells were cultured for 24 h at 37 °C. Migrated cells in the underside of the membrane were trypsinized and fluorescently stained with Calcein-AM (8 μM) for 45 min. The number of migrating cells was determined by measuring fluorescence (excitation wavelength of 485 nm and an emission wavelength of 520 nm) with a microplate reader on Infinite M200.

### 4.7. Transwell Invasion Assay

A transwell invasion assay was carried out to examine tumor invasiveness as previously described [43] with some modifications. Corning Matrigel Matrix (Corning, Tewksbury, MA, USA) was reconstituted in coating buffer 0.01 M Tris (pH 8.0) and 0.7% NaCl. One hundred µL of pre-cooled to 4 °C Matrigel was added to 8-μm transwell filters. Coated invasion chambers were plated at 37 °C for 2 h. The remaining liquid was removed. Firstly, HMVEC cells were plated (5 × 10^4^ cells/mL in the 24 well invasion chambers) in EBM medium. After 6 h, the medium was aspirated and on the top of HMVEC, SKOV-3 cells were seeded in McCoy’s 5A medium, sitagliptin (50 µM), and paclitaxel (10 nM). SKOV-3 cells were stained Calcein-AM (8 μM) for 45 min before seeding. The plates were incubated overnight in a humidified tissue culture incubator at 37 °C, in a 5% CO_2_ atmosphere. After 24 h, migrated cells were trypsinized and the fluorescence of SKOV-3 stained with Calcein-AM was measured.

### 4.8. RNA Isolation and Quantification

Cytoplasmic RNA was isolated from ovarian cancer cells cultured in 6-well plates, using the RNeasy Mini Kit (Qiagen, Germantown, MD, USA) in accordance with the manufacturer’s protocol. The purity and concentration of the isolated RNA were measured spectrophotometrically using the Infinite M200 PRO reader with NanoQuant plate (TECAN Männedorf, Switzerland). After RNA isolation, reverse transcription was performed using a High Capacity cDNA Reverse Transcription Kit with RNase Inhibitor (ThermoFisher Scientific, Waltham, MA, USA). qPCR was used to quantitatively determine the expression levels of *DPP-4* and *GAPDH* with Taq Man Gene Expression Assays (ThermoFisher Scientific, Waltham, MA, USA) in accordance with the manufacturer’s protocol using Light Cycler 480 (Roche, Burgess Hill, UK). Data were normalized based on the expression of *GAPDH* and compared using the ΔCt method. The following probes were used: DPP-4 (Hs00897386_m1) and GAPDH (Hs99999905_m1).
